# Long-term renal graft outcome after parathyroidectomy - a retrospective single centre study

**DOI:** 10.1186/s12882-020-01723-x

**Published:** 2020-02-18

**Authors:** Margret Patecki, Irina Scheffner, Hermann Haller, Wilfried Gwinner

**Affiliations:** Department of Nephrology and Hypertension, Hannover Medical School, Carl-Neuberg-Straße 1, 30635 Hannover, Germany

**Keywords:** Renal transplantation, Parathyroidectomy, Hyperparathyroidism, Hypercalcemia, Kidney function

## Abstract

**Background:**

Surgical correction of hyperparathyroidism after kidney transplantation has been associated with significant graft function decline. We examined the effects of parathyroidectomy on short- and long-term graft function and its potential predictors.

**Methods:**

For this retrospective, monocentric study we identified 48 (5.5%) out of 892 patients from our protocol biopsy program who received renal transplantation between 2000 and 2007, with parathyroidectomy after transplantation. Data from up to three years after parathyroidectomy was collected and analyzed with multivariable linear regression analyses.

**Results:**

Main indications for parathyroidectomy were hypercalcemia and graft calcifications. Parathyroidectomy was successful in 47 patients, with a median drop in serum intact parathormone (iPTH) from 394 to 21 pg/ml. Mean estimated glomerular fitration rate (eGFR) before parathyroidectomy was 60 ± 26 ml/min. At three months after parathyroidectomy, the eGFR was 46 ± 18 ml/min (*p* < 0.001) but remained stable at one and three years (50 ± 20; 49 ± 20 ml/min). The median annual eGFR change was − 0.5 ml/min before and + 1.0 ml/min after parathyroidectomy.

Multivariable modeling identified high iPTH levels and higher eGFR before parathyroidectomy as predictors of the eGFR drop after parathyroidectomy. Lower graft function twelve months after parathyroidectomy was predicted by the eGFR before and the iPTH drop after surgery.

**Conclusions:**

These results indicate that the extent of parathyroidectomy is critical and too much lowering of iPTH should be avoided by timely parathyroidectomy, before reaching extreme high iPTH values. In view of the observed loss of eGFR, parathyroidectomy can be considered safe in patients with an eGFR above 30 ml/min.

## Background

Persisting or even worsening hyperparathyroidism after kidney transplantation affects between 17 and 50% [1–5] of the transplant population and can lead to worsening graft function, bone disease and extraskeletal calcifications [1, 6–10]. Despite its off-label character, calcimimetics are increasingly used in this condition to control serum calcium and parathormone levels [11]. Nevertheless, the long-term consequences on bone metabolism are unknown [12, 13] and side effects or lacking efficacy are frequent problems. These patients may benefit from parathyroidectomy which is reported with rates of about 5% in the transplant population, thus avoiding the above mentioned complications [1, 7, 14].

Previous studies have reported differing results of parathyroidectomy after kidney transplantation. Although regarded as an efficient treatment, concerns have been raised that parathyroidectomy adversely affects the graft. In the retrospective study of Schwarz et al. a decrease in creatinine clearance by 10% was reported, without relevant recovery during a 12-month follow-up [15]. Evenepoel et al. also reported an increase in serum creatinine by 16% in the first 6 months after parathyroidectomy, with partial reversal and stabilization of graft function in the long term over 4 years [16]. In another study, patients with parathyroidectomy had 6-year graft survival of less than 15%, whereas patients without had a graft survival of approximately 70%. However, in the multivariable analysis, parathyroidectomy was not a significant factor [17].

Currently, it is not possible to predict which patient will suffer from a temporary or persistent decline in graft function after parathyroidectomy. To this end, (i) a greater iPTH drop (ii) lower serum calcium, (iii) and requirement for more intense substitution with calcium and vitamin D analogues for hypocalcemia after parathyroidectomy, (iv) lower baseline creatinine before parathyroidectomy, (v) and the time interval between parathyroidectomy and transplantation have been inconsistently reported as potential factors of a declining renal function after parathyroidectomy [3, 15, 18, 19]. Furthermore, the effects of different extents of parathyroid tissue resection on the graft function are still under discussion [15, 19, 20].

Aims of this study were to examine the effect of parathyroidectomy on the graft function and to explore potential determinants of the loss of transplant function and recovery in a well-documented patient cohort with protocol biopsies and long-term follow-up.

## Methods

### Patients

In this retrospective study, adult patients were included who received a kidney transplant alone or in combination with another solid organ at Hannover Medical School between 2000 and 2007 and who participated in our protocol biopsy program. Protocol biopsies were performed 6 weeks, 3 and 6 months after transplantation. Data were collected prior to and at the time of transplantation, at the time points of protocol biopsies and any additional biopsies, and in yearly intervals after transplantation. For patients who were followed-up elsewhere, data were retrieved by contacting their local caregivers. Data collection and analysis was performed with informed consent of the patients and with approval of the ethic board (no 2765) of the Hannover Medical School.

### Methods

Renal function was assessed by the estimated glomerular filtration rate using the Cockcroft&Gault formula. All parathormone levels were analyzed as intact PTH (normal limits: 10–65 pg/mL; Advia Centauer System, Siemens Corp., Germany). Serum calcium and phosphate levels were determined by an autoanalyzer of the hospital’s laboratory (normal ranges: 2.15–2.60 mmol/L; 0.73–1.35 mmol/L, respectively), without correcting serum calcium for albumin concentrations. Delayed graft function was defined as urine output of less than 500 ml in the first 24 h after transplantation and/or need of dialysis because of graft dysfunction within the first week after transplantation. Biopsies were evaluated according to the Banff classification. Besides routine stainings, *von Kossa* stain was performed on those cases which had tubular or interstitial crystalloid deposits [21]. Calcifications were roughly characterized as ‘mild’ with up to 2 foci of crystalloid deposits per microscopic section of the biopsy at 200fold magnification and ‘severe’ with > 2 foci. There was no specific medical treatment for calcification. Rejections were treated as reported elsewhere in detail [22].

### Statistical analysis

The IBM SPSS statistical software package version 24 was used for statistical analysis. Continuous variables with normal distribution are given as means±SD, data without normal distribution as medians. Continuous data were compared with the Kruskal-Wallis and Mann-Whitney test. The Spearman rank test was used for correlation analyses. Kaplan–Meier analysis and the log-rank test were used to compare graft survival of patients with and without parathyroidectomy. Multivariable linear regression analyses were performed to assess the effect of clinical and laboratory factors on eGFR, using backward selection and a cutoff *p* value of < 0.05. Alternative models using forward selection or no variable selection were tested in comparison. Variables chosen for the multivariable modeling were selected from Table 2, using a cutoff *p* value of < 0.15, but excluding variables with redundant information (such as serial eGFR measurements) or variables with clear causality secondary to another, significant variable (e.g. low serum phosphate caused by parathormone). Further, the large list of candidate variables shown in Table 2 and Additional file 1 was thoroughly examined for variables with potential bearing on parathyroidectomy associated adverse effects on graft, even without showing a *p* value of < 0.15. Statistical significance was assumed for *p* < 0.05.

## Results

In a cohort of 892 patients in our database who were transplanted between 2000 and 2007, 48 patients (5.4%; *n* = 23 female, *n* = 25 male) were identified with parathyroidectomy after kidney transplantation. Pretransplant and perioperative data of these patients are depicted in Table 1. Five patients had their second kidney transplantation. Three patients received combined pancreas and kidney transplantation. Delayed graft function occurred in 14 patients (29%). 40 patients (83%) received a graft from a deceased donor. Low parathormone levels in the year before transplantation, defined as parathormone levels below 2.5 times above the upper normal value were observed in only three patients. The highest eGFR within the first 6 months after transplantation was 67 ± 25 ml/min. Five patients were on therapy with cinacalcet, seven patients were treated with bisphosphonates, and one patient with calcitonin directly before parathyroidectomy.

**Table 1:** Description of patients

Age (mean ± SD)	47.7 ± 11.4
Gender (male/female)	25 / 23 (52.1 / 47.9)
Renal replacement therapy before transplantation hemodialysis / peritoneal dialysis	44 / 4 (91.7 / 8.3)
Time on dialysis (months; mean ± SD)	78.7 ± 32.8
Body mass index at transplantation (mean ± SD)	24.2 ± 3.7
Transplantation data	
Donor serum creatinine (μmol/l; mean ± SD)	80.1 ± 36.5
Donor age (mean ± SD)	45 ± 17
Donor gender (male; female)	20 (43)/ 27 (57)
Heterogeneous / homogeneous donor/recipient gender (female donor / male donor)	20 (13 / 7) / 26 (15 / 11)
Second or third kidney transplantation	5 (10.4)
Additional pancreas transplantation	3 (6.3)
Living donor transplantation	8 (16.7)
Eurotransplant Senior Program	1 (2)
Pre-formed antibodies > 0%	3 (6)
Mean number of HLA mismatches (A/B/DR)	2.21 ± 1.3
Cold ischemic time (hours; mean ± SD)	16.3 ± 9.5
Delayed graft function	14 (29.2)
CMV IgG positive recipient	26 (55)
Donor CMV IgG positive	25 (53)
Immunosuppressive therapy	
Induction therapy: IL-2 AB / ATG / none / unknown	38 / 3 / 6 / 1 (79.2 / 6.3 / 12.5 / 2.1)
Ciclosporin A	35 (72.9)
Tacrolimus	9 (18.8)
Mycophenolate mofetil	30 (62.5)
Rapamycin	4 (8.3)
Steroids	46 (95.8)
Main reason for ESRF	
Unknown	17 (35.4)
Glomerulonephritis	15 (31.3)
Tubulointerstitial disease	4 (8.3)
Hypertensive or diabetic nephropathy	3 (6.3)
Congenital disease	8 (16.7)
Other	1 (2.1)
Comorbidities before or at transplantation	
Heart failure	1 (2)
Hypertension	46 (95.8)
Peripheral arterial disease	5 (10.4)
Coronary heart disease	4 (8.3)
Stroke	1 (2.1)
Hepatitis C	2 (4.2)
Diabetes type I / II	4 (8.3) / 1 (2.1)
Hypercholesterolemia	20 (41.7)
Smoking still present / given up / never / unknown	4 / 11 / 25 / 8 (8.3 / 22.9 / 52.1 / 16.7)
Pregnancies before actual transplantation	19 (40)
Blood transfusions before transplantation	14 (30)

Proportions are depicted as number of patients, with percentages in brackets

*ATG* Anti-thymocyte globulin, *CMV* Cytomegalovirus, *ESRF* End stage renal failure, *HLA* human leukocyte antigen, *IL-2 AB* Interleukin-2 antibodies, *SD* Standard deviation

Parathyroidectomy was intended as subtotal resection in 14 cases, as total resection with auto-transplantation in 28 cases, and as total resection without auto-transplantation in 4 cases; in 2 patients further information was not available. Five patients had recurrent hyperparathyroidism after parathyroidectomy performed before transplantation. In one patient, two parathyreoidectomies after transplantation were necessary. The median time between transplantation and parathyroidectomy was 19.5 months (range: 4–80 months), with 15 patients receiving the parathyroidectomy within the first year, 17 patients within the second year, and 15 patients after the second year post-transplantation. Main indication for parathyroidectomy was repeated hypercalcemia due to hyperparathyroidism not responsive to medical management (*n* = 34; 71%). Calcification in the renal graft (in one or more preceding biopsies) in the presence of high serum calcium and/or elevated parathormone levels was another reason for parathyroidectomy in 31 (65%) patients. Calcification was observed as single finding in one biopsy in 14 patients, in two biopsies in 14 patients and in 3 biopsies in 3 patients. 19 cases had mild calcification and 8 severe calcifications and four were without grading.

**Fig. 1:**
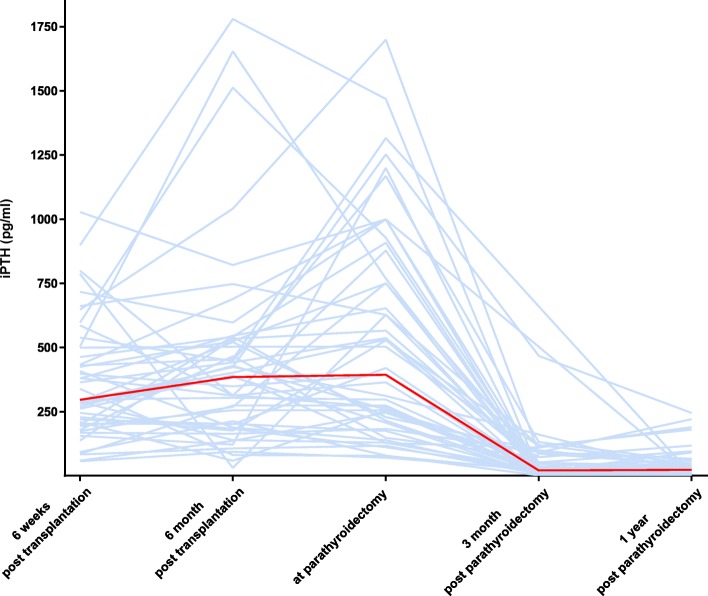
Intact parathormone levels before and after parathyroidectomy. Blue lines represent individual values; the red line represents the median course of all patients. iPTH; intact parathormone

**Fig. 2:**
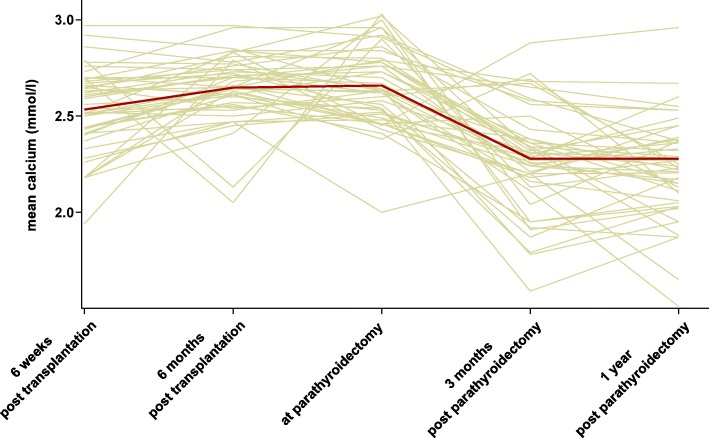
Serum calcium before and after parathyroidectomy. Green lines represent individual values; the red line represents the mean

**Fig. 3:**
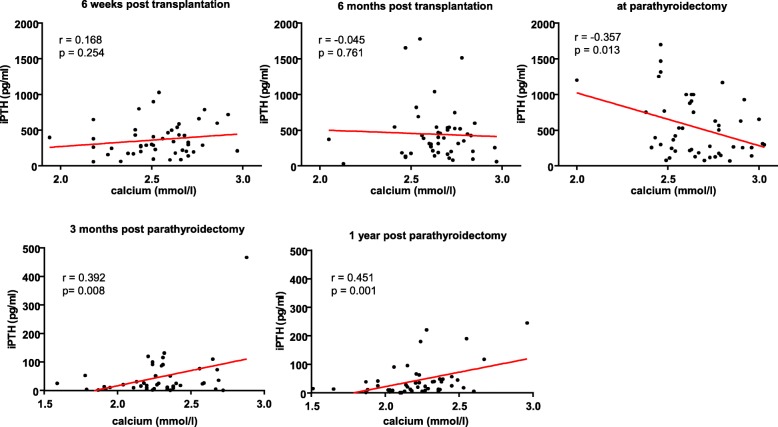
Correlation between parathormone levels and serum calcium before and after parathyroidectomy. Note different scales for iPTH values pre- and post-parathyroidectomy. iPTH; intact parathormone

The post-transplant course of parathormone and serum calcium values is shown in Figs. 1 and 2. At 6 weeks, the median iPTH was 296 pg/ml (range: 57–1028 pg/ml) and 385 pg/ml (range: 31–1780 pg/ml) at 6 months. Mean serum calcium levels at 6 weeks after transplantation were 2.53 ± 0.21 mmol/l (normal range: 2.15–2.6 mmol/l) and 2.65 ± 0.17 mmol/l at 6 months. Mean serum phosphate levels were 0.63 ± 0.29 (normal range: 0.73–1.35 mmol/l) and 0.94 ± 0.27 mmol/l at 6 weeks and 6 months post-transplantation, respectively (not shown). Directly before parathyroidectomy, median iPTH was 394 pg/ml (range: 71–1699 pg/ml), mean serum calcium 2.63 ± 0.20 mmol/l, and phosphate 0.89 ± 0.26 mmol/l. One patient had hypocalcemia due to a high dose of cinacalcet at this time. iPTH values showed a weak inverse correlation with serum calcium (r = − 0.36; *p* < 0.05) at this time. This correlation was not observed at 6 weeks and 6 months posttransplantation (Fig. 3). Serum phosphate correlated with iPTH levels, with r = − 0.33 at 6 weeks and r = − 0.32 at 6 months (p < 0.05) (not shown).

At 3 months after parathyroidectomy, iPTH values had dropped in all patients, showing a median of 21 pg/ml (range: 1–467 pg/ml) (Fig. 1). For three patients there were no iPTH values available at 3 months after parathyroidectomy. In twenty-three patients (48%) iPTH values were in the normal range of 10–65 pg/ml, in eleven patients (23%) below the lower normal, and eleven patients (23%) had iPTH values between the upper normal and 300 pg/ml. One year after parathyroidectomy, median iPTH was 24 pg/ml (range: 1–245 pg/ml) and mean serum calcium was 2.23 ± 0.26 mmol/l. Calcium and parathormone levels were moderately correlated after parathyroidectomy (Fig. 3). Serum calcium fell in most patients, with an average of 2.29 ± 0.27 mmol/l at 3 months after parathyroidectomy (Fig. 2), and phosphate rose to 1.16 ± 0.35 mmol/l. Hypercalcemia above 2.6 mmol/l was present in only 3 patients (6.3%). Hypocalcemia below 2.15 mmol/l occurred in eleven patients (23%). At one year after parathyroidectomy, 16 patients had calcium supplements, 37 patients had calcitriol or calcidiol and 2 patients colecalciferol to maintain calcium homeostasis. None of the patients received cinacalcet.

**Fig. 4:**
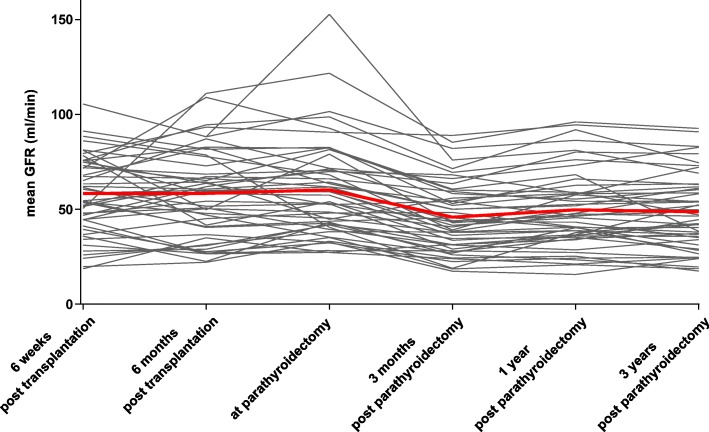
eGFR before and up to 3 years after parathyroidectomy. Black lines represent individual eGFR values, the red line the mean eGFR. *eGFR* estimated glomerular filtration rate

The changes in estimated eGFR are shown in Fig. 4. Directly before parathyroidectomy the mean eGFR was 60 ± 26 ml/min. Within the first 3 months, the eGFR dropped to 46 ± 18 ml/min (*p* < 0.001) but remained stable at one and three years after parathyroidectomy (50 ± 20; 49 ± 20 ml/min) (Fig. 4). There were no graft losses during follow-up of three years after parathyroidectomy. The median annual eGFR change was − 0.5 ml/min before and + 1.0 ml/min in the time interval between parathyroidectomy and the following 3 years. For comparison, overall 15-year death-censored graft survival and eGFR decline were similar to that of the 844 patients who had not undergone parathyroidectomy (cumulative survival of 76 vs. 71%, *p* = 0.356; median annual loss of eGFR of − 2.47 vs. -2.05 ml/min, *p* = 0.877; for patients with and without parathyroidectomy).

In a comprehensive analysis, several pre-, peri-, and posttransplant variables were explored for possible relationship with the graft function at 3 months and 12 months after parathyroidectomy. In univariable analyses (Table 2) the time interval between transplantation and parathyroidectomy and the serum calcium concentration before parathyroidectomy were not correlated with the eGFR loss (*p* = 0.183; *p* = 0.200). Younger age and higher body weight were weakly associated with a greater loss of eGFR (*p* = 0.055 and *p* = 0.065 respectively). Male patients had a greater loss of eGFR (*p* = 0.002). Calcification of the renal graft tissue was not linked with a greater loss of eGFR after parathyroidectomy (− 16.45 ml/min compared with − 8.8 ml/min in patients without calcifications, *p* = 0.461). Also, severity and frequency of calcification findings were not associated with the loss of eGFR (*p* = 0.266 and 0.589 respectively). Interestingly, we also did not observe an association of the frequency or severity of calcification findings with interstitial fibrosis and tubular atrophy or with arteriolar hyalinosis (*p* < 0.539 and *p* < 0.821). Multivariable analysis identified higher eGFR and iPTH values directly before parathyroidectomy as predictors of a greater loss of eGFR at 3 months after parathyroidectomy (R = 0.625) (Table 3a). Alternative models without variable selection or with less stringent cutoffs for selection had lower or not relevantly higher R values and showed no changes in the ß-coefficient of the significant variables (not shown). Variables that were not significant in univariate analyses and not considered in the different multivariable models were shown in an additional file (see Additional file 1).

**Table 2:** Correlation of the GFR-change with pretransplant, peri- and posttransplant clinical factors which were considered as candidate variables in the multivariable modeling

	eGFR change at 3 months post para-thyroidectomy	P value	eGFR change at 12 months post para-thyroidectomy	P value
Recipient at transplantation				
Age	0.281	0.055	0.153	0.303
Gender (male/female)	−19.40; −8.22	0.002	12.93; −6.83	0.053
Hyperparathyroidism before transplantation, *yes; no*	−14.10; −21,54	0.414	−10.59; −13.93	0.414
Parathyroidectomy before transplantation, *yes; no*	−16.87; −11.29	0.568	−14.78; −9.88	0.158
Time on dialysis before transplantation	−0.078	0.603	− 0.195	0.189
Re-transplanted patients, 2nd or 3rd transplantation; 1st transplantation	−20.34; − 11.68	0.372	−13.93; − 10.23	0.535
Body weight at transplantation	−0.277	0.065	−0.265	0.078
Body mass index at transplantation	−0.146	0.340	−0.239	0.114
Early post-transplantation course				
Delayed graft function, *yes; no*	−12.42; −16;57	0.924	−10.23; −11.76	0.505
Best eGFR in the first 6 weeks post- transplantation	−0.542	< 0.001	−0.239	0.055
Post-transplant-related factors				
Last serum calcium before parathyroidectomy	0.190	0.200	0.290	0.048
Mean serum calcium within the first 6 months after transplantation	0.143	0.338	0.220	0.137
Last serum phosphate before parathyroidectomy	−0.034	0.832	−0.132	0.412
Mean serum phosphate within the first 6 months after transplantation	0.031	0.836	−0.115	0.441
Last iPTH before parathyroidectomy	−0.226	0.127	−0.409	0.004
Mean iPTH within the first 6 months after transplantation	−0.146	0.328	−0.278	0.058
iPTH change after parathyroidectomy	0.199	0.190	0.374	0.011
Months between transplantation and parathyroidectomy	0.198	0.183	0.193	0.194
eGFR 6 weeks after transplantation	−0.464	0.001	− 0.207	0.162
eGFR 3 months after transplantation	−0.524	< 0.001	− 0.227	0.124
eGFR 6 months after transplantation	− 0.373	0.010	−0.129	0.386
eGFR 3 months before parathyroidectomy	−0.501	< 0.001	−0.361	0.013
Last eGFR before parathyroidectomy	−0.500	< 0.001	−0.398	0.006
eGFR change before parathyroidectomy	−0.050	0.741	−0.159	0.285
Nephrocalcinosis at biopsy, *yes; no*	−16.45; −8.8	0.461	−11.5; −7.9	0.242
Rejections until parathyroidectomy *yes; no*	−14.2; −10.5	0.749	−7.7; − 11.3	0.701

The change in eGFR 3 months and 12 months after parathyroidectomy was calculated individually for each patient as the difference between the post-parathyroidectomy value and the eGFR before parathyroidectomy, with negative values representing a loss of eGFR. PTH change: decrease in iPTH is defined as negative value. Shown are r values for continuous factors and grouped medians for categorical variables. *eGFR* estimated glomerular filtration rate, *iPTH* intact parathormone

**Table 3:** Factors predictive of eGFR loss at 3 (A) and 12 (B) months after parathyroidectomy

(A) Model for eGFR loss at 3 months Overall fit: R = 0.625	Univariable linear regression			Multivariable linear backward stepwise regression		
	ß	CI 95%	p value	ß	CI 95%	p value
Age at transplantation (years)	0.305	0.038 0.573	0.026			
Female gender	10.046	4.460 15.632	0.001			
Body weight at transplantation (kg)	−0.205	−0.419 0.009	0.060			
Last iPTH before parathyroidectomy (pg/ml)	−0.005	−0.013 0.002	0.165	−0.006	− 0,012 0.000	0.043
Time between parathyroidectomy and transplantation (months)	0.114	−0.094 0.321	0.275			
Last eGFR before parathyroidectomy (ml/min)	−0.280	−0.399 -0.160	0.000	−0.288	− 0.404 0.172	0.000
(B) Model for eGFR loss at 12 months Overall fit: R = 0.545	Univariable linear regression			Multivariable linear backward stepwise regression		
	ß	CI 95%	p value	ß	CI 95%	p value
Female gender	4.866	−0.556 10.288	0.077			
Body weight at transplantation (kg)	−0.163	−0.354 0.027	0.092			
Parathyroidectomy before transplantation	−5.522	−15.444 4.400	0.268			
Last iPTH before parathyroidectomy (pg/ml)	−0.008	−0.014 -0.002	0.016			
iPTH change after parathyroidectomy (pg/ml)	0.007	0.000 0.014	0.043	0.009	0.002 0.015	0.008
Last serum calcium before parathyroidectomy (mmol/l)	12.473	−1.336 26.282	0.076			
Last eGFR before parathyroidectomy (ml/min)	−0.178	−0.296 -0.060	0.004	−0.193	−0.304 -0.082	0.001

The change in eGFR after parathyroidectomy was calculated individually for each patient as the difference between the post-parathyroidectomy value and the eGFR before parathyroidectomy, with negative values representing a loss of eGFR

The overall fit of the models is given in the left upper column head

iPTH change: decrease in iPTH is defined as negative value

*CI* Confidence interval, *eGFR* estimated glomerular filtration rate, *iPTH* intact parathormone

Recovery from the observed loss in graft function determines the longterm outcome. Therefore, we examined factors which determine the lower graft function one year after parathyroidectomy. In univariable analyses (Table 2), most factors were comparable with the 3 months results, including male gender (*p* = 0.053), body weight (*p* = 0.078), iPTH and eGFR before parathyroidectomy (*p* = 0.004 and *p* = 0.006, respectively). In addition, the decrease of iPTH after parathyroidectomy was a significant factor (*p* = 0.011). The multivariable analysis (Table 3b) identified higher eGFR before and greater iPTH drop after parathyroidectomy as a predictor of a greater loss in eGFR at 12 months (R = 0.545). Similarly, as described above alternative models did not show improved prediction of eGFR.

## Discussion

Persistent Hyperparathyroidism is frequent after kidney transplantation, with approximately 17–50%, mostly occurring as tertiary hyperparathyroidism [1–5]. Tertiary hyperparathyroidism represents a relevant clinical problem due to adverse effects of hypercalcemia in terms of extraosseous calcification and worsening of graft function [1, 6–10]. In this study, the main indications for parathyroidectomy were hypercalcemia which was present in 34 of 48 patients (71%) and calcification of the renal graft (*n* = 31). Parathyroidectomy was successful in the majority of patients. Only one patient had to undergo re-parathyroidectomy and 3 patients had serum calcium levels above the upper normal value after surgery.

The adverse short-term effect of parathyroidectomy on renal graft function is well-known [15, 16, 19]. We were interested in the longterm graft function of patients with parathyroidectomy and the factors that determine this outcome.

Renal graft function in the whole group of patients appeared to be relatively stable before parathyroidectomy with an eGFR slope of − 0.5 ml/min*year. This loss is less than reported in studies that analyzed graft function over longer follow-up periods after transplantation, with an annual GFR decline of 1.1–1.7 ml/min [23, 24]. However, most patients were in an early period after transplantation in which gain of graft function is usually observed within the first year (unpublished data). Also, parathormone has been suggested as a driver of glomerular filtration [25–29] which may have obscured a decline in GFR. After parathyroidectomy, a uniform and significant decrease in eGFR by 25% was observed. This loss of eGFR is greater than reported in other studies showing a 10% decline in eGFR [16, 19]. The course of renal function after the initial loss of eGFR is unknown. Our study clearly shows that renal graft function stabilizes in patients with parathyroidectomy. Over the course of three years, the median eGFR was comparable with the eGFR three months after parathyroidectomy. Moreover, individual calculation of the eGFR slope before and after surgery showed that the eGFR slope changed to positive values after parathyroidectomy, with an average increase of 1.0 ml/min*year, which could reflect an improvement or recovery after correction of hyperparathyroidism. Yet, because of the substantial initial drop of eGFR, complete recovery of renal function to pre-parathyroidectomy eGFR values was not observed. Despite this incomplete recovery, graft survival and annual eGFR loss over the entire course of 15 years after transplantation was comparable with patients who had no parathyroidectomy after transplantation. Subanalysis of patients with renal graft calcification showed that there is no difference in the initial drop in eGFR after parathyroidectomy and the further course of graft function compared with patients without graft calcification. Due to the lack of systematic re-biopsies we were not able to determine whether calcifications decrease after normalization of the calcium-phosphate metabolism. Also, no systematic study was possible by biopsies performed after parathyroidectomy to associate the functional impairment with histomorphological changes.

Pre-parathyroidectomy parathormone concentration, glomerular filtration rate, serum calcium levels and the time between transplantation and parathyroidectomy have been proposed as important influencing factors for the eGFR loss after parathyroidectomy. We used the comprehensive documentation of clinical, laboratory and biopsy data of this patient cohort to explore if further factors determine the magnitude of eGFR loss after parathyroidectomy. Our univariable analyses largely confirmed the reported associations and identified only gender as an additional factor. The time point of parathyroidectomy was not a relevant factor in the present time range of 4–80 months posttransplantation. The small number of patients precluded establishing separate models for predicting the eGFR loss in subgroups with different time periods between parathyroidectomy and transplantation. However, univariate sensitivity analyses showed that patients with parathyroidectomy beyond the second year after transplantation had a lower eGFR before parathyroidectomy and a smaller loss of eGFR after parathyroidectomy, compatible with our multivariable model (not shown). Further, neither the univariate analysis (Table 2) nor the multivariable modeling (Table 3) identified the timing of parathyroidectomy as a significant factor. Therefore, parathyroidectomy may be decided at any time when conservative therapy fails and thus an indication for surgical therapy is present. Linear regression analysis showed that the loss in eGFR is significantly determined by the renal graft function and the serum parathormone concentration immediately before parathyroidectomy. The outcome at one year was best predicted by the eGFR before parathyroidectomy and the drop in parathormone by the surgery.

We recognize the limitations of our study which is retrospective and cannot prove causality of the observed associations. Results may not directly applicable to transplant settings in other centers and not to all patients with potential indication for parathyroidectomy as our study included only forty-eight patients.

## Conclusions

Several conclusions can be drawn from our results. First, because the average eGFR loss was 14 ml/min, indication for parathyroidectomy can be considered safe only in patients with an eGFR above 30 ml/min because otherwise, expected eGFR after parathyroidectomy will be in the range of stage V of chronic kidney disease. This is important because renal function is a highly significant factor of patient and graft survival [22, 30]. Second, as the magnitude of iPTH elevation before parathyroidectomy and the drop in iPTH values after parathyroidectomy are major determinants of the loss in eGFR, timely parathyroidectomy –before reaching extreme high iPTH values– may be beneficial, thus avoiding the adverse effect of a steep drop in iPTH. Also important in this line, an appropriate extend of parathyroidectomy could help to avoid a steep drop in iPTH after surgery. In our patient group, 23% of patients experienced hypocalcemia and 23% had serum parathormone concentrations below the lower normal limit, implicating that a substantial proportion probably had an inordinate removal of parathyroid tissue. This point is also highlighted by the fact that one third of patients required calcium supplementation one year after parathyroidectomy and 77% were treated with vitamin D compounds. Intraoperative iPTH monitoring could help to avoid an excessive parathyroid tissue removal that can lead to too low iPTH levels after surgery [31].

It should be noted that current guidelines do not give specific recommendations regarding target iPTH values after parathyroidectomy, the extent of parathyroid tissue removal and its timing after kidney transplantation [32–34]. For patients with chronic kidney disease of all stages but without dialysis treatment, iPTH concentrations within the normal range were suggested. Lowering of iPTH values below the lower normal limit as present in a substantial proportion in our patients should be certainly prevented.

## Supplementary information

**Additional file 1.** Correlation of the GFR-change with clinical factors. The table displays the variables that were not significant in univariate analyses and not considered in the different multivariable models.

## Data Availability

The datasets used and/or analysed during the current study are available from the last author (Gwinner.Wilfried@mh-hannover.de) on reasonable request.

## Abbreviations

ATG: anti-thymocyte globulin
CI: confidence interval
CMV: cytomegalovirus
eGFR: estimated glomerular filtration rate
ESRF: end stage renal failure
HLA: human leukocyte antigen
IL-2 AB: interleukin-2 antibodies
iPTH: intact parathormone
SD: standard deviation
