# Supplementary material for: Long-term renal graft outcome after parathyroidectomy - a retrospective single centre study
Source: BMC Nephrol. 2020 Feb 18;21:53. doi: 10.1186/s12882-020-01723-x (PMC7027287; doi:10.1186/s12882-020-01723-x)
Supplement: Supplementary file 1 — Additional file 1. Correlation of the GFR-change with clinical factors. The table displays the variables that were not significant in univariate analyses and not considered in the different multivariable models. [file 12882_2020_1723_MOESM1_ESM.pdf]

Additional file 1

Correlation of the GFR-change with clinical factors. The table displays the variables that were not significant in univariate analyses and not considered in the different multivariable models.

	eGFR change at 3 months post para-thyroidectomy	P value	eGFR change at 12 months post para-thyroidectomy	P value
**Renal replacement therapy before transplantation** *hemodialysis / peritoneal dialysis*	-14.25; -9.4	0.326	-10.6; -8.9	0.727
Main reason for endstage renal failure (%)		0.891		0.849
glomerulonephritis/vasculitis	-9.61		-11.31	
tubulointerstitial diseases	-13.92		-11.77	
hypertensive/diabetic nephropathy	-17.77		-2.85	
congenital disease	-9.80		-8.01	
Co-morbidity before and at transplantation (%)				
Heart failure, *yes; no*	-5.37; -14.10	0.478	1.65; -10.59	0.304
Coronary heart disease, *yes; no*	-10.98; -14.10	0.568	-4.64; -10.59	0.290
Peripheral arterial disease, *yes; no*	-17.77; -10.55	0.372	-13.93; -8.94	0.491
Type I diabetes, *yes; no*	-19.71; -10.55	0.186	-9.15; -10.23	0.465
Type II diabetes, *yes; no*	-23.19; -10.55	0.326	-16.50; -10.23	0.836
CMV IgG, *positive; negative*	-10.45; -15.29	0.494	-7.61; -11.31	0.294
Pregnancies before actual transplantation, *yes; no*	-7.55; -9.80	0.962	-6.83; -6.62	0.718
Blood transfusions before transplantation, *yes; no*	-10.55; -14.10	0.630	-10.69; -7.34	0.778
Hypertension	-13.54	0.895	-10.59	0.599
Hepatitis C	-16.45	0.668	-15.32	0.244
Hypercholesterinemia	-11.29	0.430	-10.79	0.784
**Transplant-related factors**				
Eurotransplant Senior Program, *yes; no*	-3.30; -13.83	0.426	-6.08; -10.69	0.596
Combined kidney/pancreas transplantation, *yes; no*	-21.67; -12.42	0.262	-2.85; -10.69	0.440
Pre-formed antibodies, >0%; <=0%	0.35; -14.10	0.209	-7.34; -10.59	0.801
Donor age (years)	0.161	0.284	-0.093	0.539
Donor gender *male; female*	-9.71; -14.10	0.835	-10.23; -7.88	0.711
Donor type *deceased; living*	-13.55; -16.29	0.667	-10.59; -11.66	0.627
Donor CMV IgG *positive; negative*	-9.61; -15;29	0.326	-9.88; -11.76	0.294

Donor serum creatinine (µmol/L)	-0.017	0.927	-0.106	0.474
Mean number of HLA mismatches				
HLA A mismatches	-0.006	0.966	0.104	0.486
HLA B mismatches	0.081	0.588	0.110	0.460
HLA DR mismatches	0.060	0687	0.077	0.606
Cold ischemic time (hours)	0.071	0.635	0.015	0.920
**Initial immunosupressive therapy (%)**				
Induction therapy		0.562		0.141
Anti-thymocyte globulin	-17.16		-17.45	
Interleukin-2 antibodies	-11.29		-8.01	
none	-8.58		-8.26	
other / unknown	-13.55		-10.92	
Cyclosporine A, *yes; no*	-12.42; -15.29	0.721	-7.95; -11.90	0.318
Tacrolimus, *yes; no*	-9.61; -13.83	0.514	-10.59; -9.40	0.700
Mycophenolate mofetil, *yes; no*	-14.77; -8.84	0.259	-11.11; -8.01	0.690
Rapamycin	-5.76	0.290	-8.44	0.985
Steroids	-14.10	0.337	-10.59	0.668
**Biopsy findings**				
interstitial fibrosis and tubular atrophy (%)	0.093	0.539	-0.025	0.871
arteriolar hyalinosis *yes; no*	-8.8, -14.6	0.821	-7.9; -11.1	0.330
Severity of calcification findings	-0.166	0.266	-0.179	0.230
Frequency of calcification findings	-0.081	0.589	-0.200	0.177

CMV; cytomegalovirus, HLA; human leukocyte antigen.
